# Swimming by Spinning: Spinning‐Top Type Rotations Regularize Sperm Swimming Into Persistently Progressive Paths in 3D

**DOI:** 10.1002/advs.202406143

**Published:** 2024-12-18

**Authors:** Xiaomeng Ren, Hermes Bloomfield‐Gadêlha

**Affiliations:** ^1^ School of Engineering Mathematics and Technology & Bristol Robotics Laboratory University of Bristol Bristol BS8 1UB UK

**Keywords:** 3D head orientation, cell translations and rotations, flagellar asymmetry detection, flagellar beating asymmetry, free sperm swimming in 3D

## Abstract

Sperm swimming is essential for reproduction, with movement strategies adapted to specific environments. Sperm navigate by modulating the symmetry of their flagellar beating, but how they swim forward with asymmetrical beats remains unclear. Current methods lack the ability to robustly detect the flagellar symmetry state in free‐swimming spermatozoa, despite its importance in understanding sperm motility. This study uses numerical simulations to investigate the fluid mechanics of sperm swimming with asymmetrical flagellar beats. Results show that sperm rotation regularizes the swimming motion, allowing persistently progressive swimming even with asymmetrical flagellar beats. Crucially, 3D sperm head orientation, rather than the swimming path, provides critical insight into the flagellar symmetry state. Sperm rotations during swimming closely resemble spinning‐top dynamics, with sperm head precession driven by the helical beating of the flagellum. These results may prove essential in future studies on the role of symmetry in microorganisms and artificial swimmers, as body orientation detection has been largely overlooked in favor of swimming path analysis. Altogether, this rotational mechanism provides a reliable solution for forward propulsion and navigation in nature, which would otherwise be challenging for flagella with broken symmetry.

## Graphical abstract

This article looks at how sperm swim forward even with uneven movements in their tail, using a virtual sperm model to show that their rotation helps keep them moving straight. It highlights that the way sperm rotate in 3D holds important clues about their tail beating, offering new insights into how cells move and how artificial swimmers could be designed.

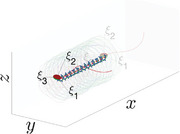

## 1 Introduction

**Figure 1:**
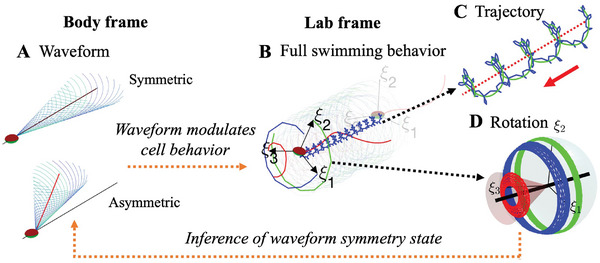
Outline of research focus: The flagellar waveform in the body frame controls sperm swimming behavior in the lab frame, enabling the detection of waveform symmetry (see Table 2 and Methods Section for definitions used in this work). A) Visualization of 3D symmetric and asymmetric waveforms in the body frame. The red line indicates the average flagellum shape, while the black line shows the sperm head's longitudinal axis. Symmetry is identified by the alignment of these lines, whereas asymmetry is shown by their offset. The head is color‐coded for clarity, and the flagellum's blue‐to‐green gradient represents time evolution over one beat cycle. B) Full swimming behavior in the lab frame, generated by symmetric or asymmetric waveforms. The blue curve traces the head's center trajectory, and the three head basis vectors, $\xi_{1,2,3}$, are shown in blue, green, and red, respectively. Snapshots of the sperm at the start and end points help visualize the overall swimming pattern. C) Lab frame trajectory of the sperm head center. The blue line shows the raw trajectory, the green line represents the smoothed average path, and the red dotted line (with a red arrow) indicates the progressive swimming axis. D) Head orientation orbits, depicting sperm head rotational movement through the orbits of the three basis vectors $\xi_{1,2,3}$. The black line represents the head precession axis, around which these orientation orbits revolve. The translucent cone shows the average tilt angle, which can reveal waveform asymmetry (Table 2).

Sperm swimming is essential for reproduction across almost all organisms, with their structure and movement adapted to the specific environments of fertilization.^[^ ^1^, ^2^, ^3^ ^]^ In internal fertilizers, like mammals, sperm navigate through the female reproductive tract to reach and fertilize the egg.^[^ ^4^, ^5^, ^6^ ^]^ External fertilizers, such as in aquatic species, rely on sperm quickly locating the egg in water, often with a limited time before losing motility due to osmotic shock.^[^ ^7^, ^8^, ^9^ ^]^ Internal fertilizers have streamlined structures with energy‐rich midpiece to penetrate viscous mucus environments,^[^ ^3^, ^10^, ^11^ ^]^ while external fertilizers exhibit simpler designs suited for aquatic fertilization.^[^ ^4^, ^12^, ^13^ ^]^ Species‐dependent adaptations result in diverse swimming strategies.^[^ ^1^, ^12^, ^14^, ^15^, ^16^, ^17^, ^18^, ^19^ ^]^ Sperm steering and navigation control, however, is achieved by modulating the symmetry state of the flagellum beating pattern in response to environmental cues.^[^ ^20^, ^21^, ^22^ ^]^ Asymmetric beating modulation is critical throughout fertilization, including processes like capacitation, hyperactivation, signaling, and chemotaxis.^[^ ^4^, ^23^, ^24^, ^25^, ^26^ ^]^ Structurally, asymmetry is integral to the sperm flagellum, from the centriole scaffold to dynein arrangement and ion channel distribution.^[^ ^27^, ^28^, ^29^, ^30^, ^31^, ^32^, ^33^, ^34^ ^]^ The flagellar symmetry state is also crucial for identifying different physiological states of the sperm flagellum and understanding how its structure drives function during sperm swimming.^[^ ^6^, ^29^, ^30^, ^35^ ^]^ Despite its fundamental importance, no robust method currently exists to measure waveform symmetry in free‐swimming sperm.^[^ ^6^, ^36^, ^37^, ^38^ ^]^ This is surprising, as a strong correlation between swimming patterns and waveform symmetry would be expected—after‐all sperm swimming directly results from flagellar motion. However, as we demonstrate in this paper, the relationship is more complex than anticipated. This work aims to investigate how the flagellar beating symmetry state manifests in swimming behavior and the challenges in detecting it (**Figure** **1**).

**Figure 2:**
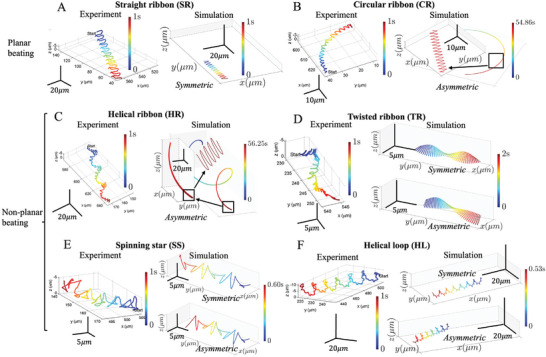
Comparison of experimental sperm trajectories with corresponding numerical reconstructions. Six representative modes of sperm head tracks in the lab frame are illustrated, with experimental baselines on the left from Ref. [44], and numerical reconstructions on the right generated by our models. A–F) depict trajectory modes of straight ribbon (SR), circular ribbon (CR), helical ribbon (HR), twisted ribbon (TR), spinning star (SS), and helical loop (HL), respectively. Time progression is represented by color gradients. The simulated results are scaled according to the bovine sperm arc length of 65 µm^[^ ^44^, ^49^, ^50^, ^51^, ^52^ ^]^ and the flagellar beating frequencies from Ref. [44]. The axis scales in simulations are matched to the experimental data, and for CR and HR modes, where the trajectory span is larger, adjusted scales are shown in the insets. For certain modes, such as SR, CR, and HR, the characteristic trace patterns can only be generated by either symmetric or asymmetric virtual models, while other modes can be reproduced by both. Trajectory classification can be found in the Methods Section. In Figure S1 (Supporting Information), all simulated trajectory patterns are depicted at the same spatial scale for comparison purposes. Reproduced with permission.^[^ ^44^ ^]^ Copyright 2017, Springer Nature.

It is generally accepted that symmetrical beating leads to progressive swimming trajectories, whilst asymmetrical waveform results in curved and circular swimming paths, leading to non‐progressive swimming^[^ ^36^, ^39^, ^40^, ^41^, ^42^ ^]^ (**Figure** **2**). In an apparent contradiction, 3D sperm tracking experiments (Figure 2) show that the majority of sperm has progressive swimming with a globally straight forward direction.^[^ ^43^, ^44^, ^45^ ^]^ Figure 2C–F (left column) depicts four representative types of sperm trajectories^[^ ^44^ ^]^ exhibiting progressive swimming: helical ribbon (HR), twisted ribbon (TR), spinning star (SS) and helical loop (HL), representing 91.7% of a total of 2133 bovine sperm (trajectory classifications are provided in Methods Section). It has long been hypothesized that, despite waveform asymmetry, global progressive swimming is enabled by the out‐of‐plane flagellar motion, which drives sperm rolling as it swims^[^ ^26^, ^37^, ^46^, ^47^, ^48^ ^]^—though the exact mechanisms by which this occurs remain unexplored.^[^ ^37^ ^]^ Furthermore, if progressive swimming can be achieved regardless of the symmetry state of the beating, how asymmetry manifests in cell swimming remains unclear. Here, we test above hypothesis (Figure 1) using mathematical modeling and simulation of free‐swimming sperm driven by a symmetric and asymmetric beating flagellum in 3D (Figure 1A) to unveil the complex relationship between the symmetry state of the beating and swimming behaviours (Figure 1B–D). This reveals a novel spinning‐top‐like motion that regularizes sperm swimming into persistently progressive swimming paths in 3D, with important consequences on empirical detection of flagellar beating symmetry.

Numerical simulations in Figure 2 and Figure S1 (Supporting Information) show that both symmetric and asymmetric waveforms can qualitatively recapitulate the diversity of progressive swimming modes in 3D sperm experiments,^[^ ^44^ ^]^ in agreement with earlier studies.^[^ ^26^, ^48^, ^53^, ^54^ ^]^ The persistent progressive swimming in 3D (Figure 2) is also consistent with recent research by Zaferani et al.,^[^ ^25^, ^37^ ^]^ which demonstrates that progressive swimming is possible with asymmetric, though non‐planar, beating. This was attributed to the potential counteraction of sperm rolling [*ibid*]. This contrasts with asymmetric but planar waveforms, which always result in curved, non‐progressive trajectories^[^ ^36^ ^]^ (Figure 2, CR). Numerical simulations in Figure 2 and Figure S1 (Supporting Information) show that the symmetry state of the flagellum does not directly manifest in sperm trajectories (compare the simulations for symmetric and asymmetric cases in Figure 2 and Figure S1, Supporting Information). Thus detection of beat symmetry from progressive swimming in 3D is challenging, if ever possible. As we further demonstrate in this study, the symmetry state cannot be inferred from 3D flagellar tracking of free‐swimming sperm in the lab frame of Refs. [55, 56, 57, 58] — as more information is necessary to distinguish beating symmetry. In all, given the persistent progressive swimming in 3D, how does waveform symmetry affect sperm motion, and how can this be experimentally detected (Figure 1)? It is implausible that all asymmetry is simply “filtered out” by sperm rolling motion, as current knowledge suggests. Rather, waveform asymmetry must be manifested and detectable at some level during cell swimming (Figure 1C,D). Indeed, we find that waveform asymmetry drives sperm into complex rotational orbits in 3D (Figure 1D), with a surprising correspondence with spinning‐top rotations.

Figure 1 illustrates the main goal of this work: to explore how the asymmetry of the flagellar beat influences sperm swimming behavior. This behavior is investigated using two key sperm motion types: sperm translations, which track the movement of the sperm head along a path (head trajectories ‐ Figure 1C), and body rotations, which track how the sperm head changes its orientation during swimming (head orientations ‐ Figure 1D). Traditional methods typically focus on the overall swimming path or trajectory of the sperm. However, as we will show, these paths alone do not provide enough information to detect the asymmetry in the flagellar beat. Instead, the key to identifying this asymmetry lies in analyzing how the sperm's body rotates and changes orientation during swimming. This rotational data reveals the subtle effects of asymmetry that are not apparent from the trajectory alone, highlighting key details that Computer‐Assisted Semen Analysis (CASA) systems, which evaluate sperm motility using 2D projections, may miss in their assessments.

## 2 Results

We conducted microhydrodynamic simulations of free sperm swimming to elucidate the role of waveform symmetry on 3D sperm motion (Figure 1). Sperm swimming behavior in the lab frame is characterized by sperm translations (head trajectories) and sperm rotations (head orientations), and the results are presented accordingly below (Figure 1). We first examine how waveform symmetry affects sperm translations (Sections 2.2, 2.3, and 2.4), then explore its impact on head rotations (Sections 2.3 and 2.4), and finally combine both to assess the impact of these findings on two common detection methods (Section 2.5): direct waveform tracking in the lab frame and sperm swimming characteristics derived from Computer‐Assisted Semen Analysis (CASA) systems,^[^ ^59^ ^]^ commonly used in clinical settings. We begin in the next section by introducing the parametric forms of the symmetric and asymmetric waveform models used in this study. The numerical implementation, concepts, and definitions used in this work are described in the Methods Section and summarized in Table 2.

**Table 1:** Comparison of simulated and experimental sperm dynamics for the HL trajectory mode.

Data source	Curvilinear velocity [µm/ s]	Helix radius (r) [µm]	Helix pitch (P) [µm]	Period of helix revolution [s]
Numerical (xyz‐model (*k* = 2π))^a)^	43.0–167.6	2.1–2.4	4.3–6.1	1.7–8
Numerical (κ‐model (*k* = 2π))	43.6–96.8	2.2–2.5	2.5–10.5	0.3–2.7
Experimental^[^ ^43^ ^]^	68.8–129.4	1.1–2.1	2.7–4.7	2.2–11.4

^a)^ The raw numerical data is dimensionless such that sperm flagellum length is taken as 1 and time metric is beat cycle. To dimension the dynamic results, the length and beat frequency of human sperm appendage are taken as 55 µm and 10–20 Hz, respectively.^[^ ^4^ ^]^

### 2.1 Symmetric and Asymmetric Flagellar Waveform Models

We focus our study on helicoidal waveforms experimentally described in the literature.^[^ ^6^, ^26^, ^60^, ^61^, ^62^ ^]^ Direct experimental observations of flagellar beating relative to the sperm head so far are limited to tethered sperm^[^ ^6^, ^24^, ^35^, ^60^ ^]^ or for 2D swimming cells.^[^ ^14^, ^36^, ^38^ ^]^ As such, the 3D beating is approximated by an elliptical helicoidal waveform, as inferred from experiments^[^ ^18^, ^60^, ^61^, ^63^ ^]^ and which has long history of usage in the literature.^[^ ^18^, ^26^, ^54^, ^62^, ^64^ ^]^ While these general helicoidal forms could theoretically generate a wide range of waveforms, doing so would risk straying from biologically plausible, species‐specific behavior, so we have avoided such extrapolation. The waveform models we consider are primarily observed in mammalian species and sea urchins in low‐viscosity environments.^[^ ^6^, ^26^, ^60^, ^61^, ^62^ ^]^ However, given the wealth of 3D beating asymmetries, 3D flagellar tracking, and 3D sperm swimming behavior in the literature, we focus this study on waveforms associated with mammalian sperm in low viscosity.^[^ ^35^, ^38^, ^44^, ^58^, ^61^, ^65^ ^]^ Mathematical description, estimations of parameter values, and the previous works that our numerical simulations are built on can be found in the Methods Section.

**Figure 3:**
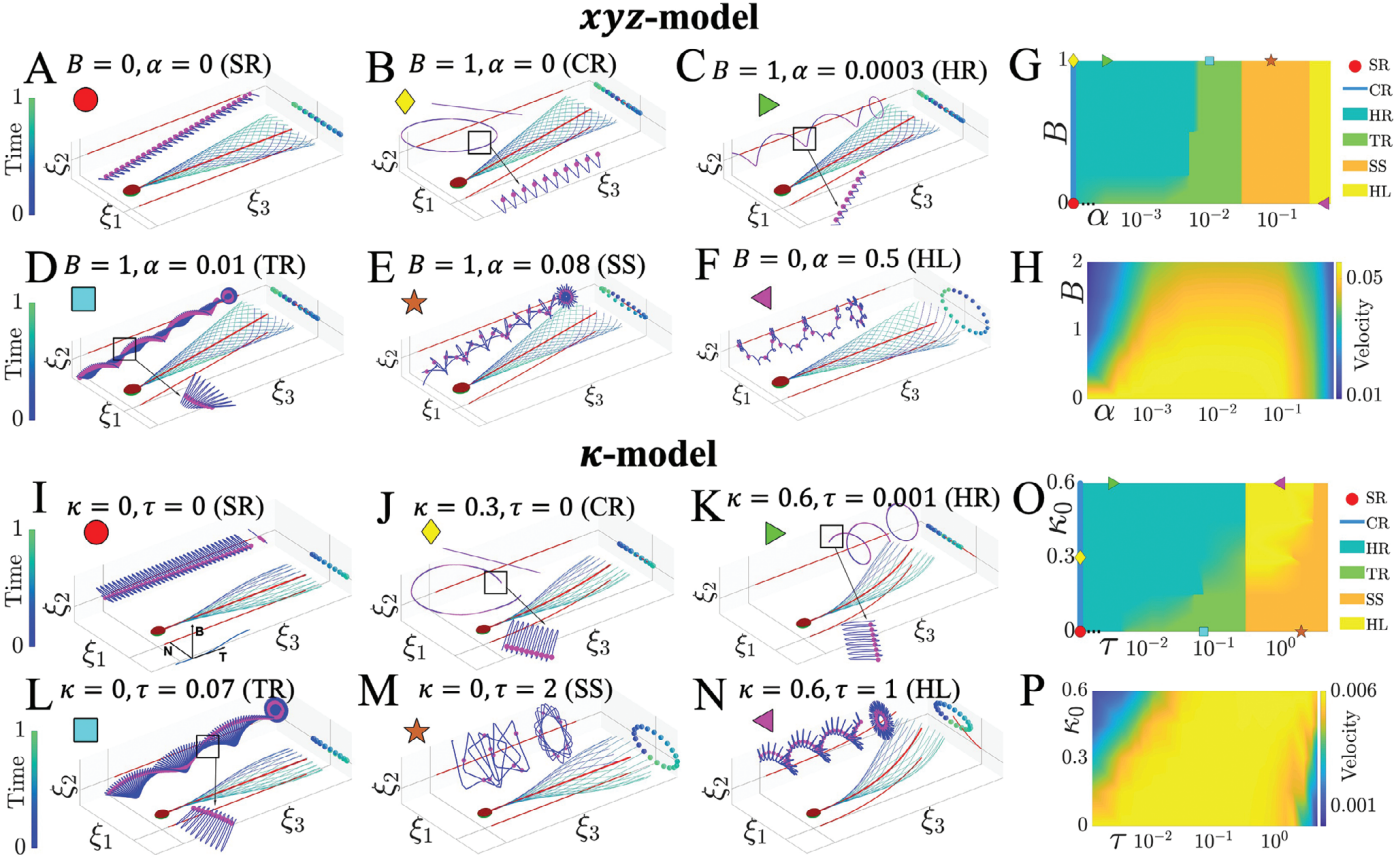
Virtual sperm models, lab trace classification, and progressive swimming velocity. Flagellum waveforms relative to the body frame are shown for the *xyz*‐model (A–F)) and the κ‐model (I–N)), alongside the corresponding lab trajectories of the sperm head center, indicated by various markers. The two key waveform factors, symmetry and rotation amplitude, are represented by the average flagellum shape (in red) on the ξ_1_ξ_3_ plane and the point clouds tracking projections of the end flagellar point on the ξ_1_ξ_2_ plane, respectively. Virtual model parameters are listed at the top of each figure. Flagellum positions throughout one beat cycle are color‐coded, and magenta points in the trajectory insets denote beat cycle intervals, highlighting the synchrony between waveform and resulting path. Six representative trajectory modes, influenced by waveform asymmetry and out‐of‐plane factors, are displayed in the parameter space in G) and O), for the *xyz*‐model with *k* = 2π and the κ‐model, respectively. Zero values of α (τ) are included in G) and O) as the limiting cases, overlaid on the logarithmic scale to show the trace modes SR and CR. The virtual model examples in A–F) and I–N) are highlighted with matching markers. H) and P) show the velocity of the lab frame trajectory along the progressive axis for the *xyz*‐model with *k* = 2π and the κ‐model, respectively. As for G) and O), the zero origins in H) and P) pertain to the vertical axis of *B* (κ_0_), and not the logarithmic horizontal axis representing α (τ)—zero is not displayed in horizontal axis. The linear speed is dimensionless, expressed in units of flagellar arc length per beat cycle. Trajectory and velocity quantifications are described in Methods Section.

Here we consider two sources of static waveform asymmetry that have been observed experimentally^[^ ^6^, ^14^, ^25^, ^35^, ^36^, ^37^, ^38^ ^]^: (1) a one‐sided bias of the waveform relative to the orientation of the sperm head long axis,^[^ ^35^ ^]^ due to internal asymmetries of the basal body and centriole, and (2) an asymmetric mean curvature of the flagellum over beat cycle.^[^ ^6^, ^14^, ^36^, ^38^ ^]^ Mathematically, these two waveform asymmetries are considered as follows: (a) a waveform side‐shift of an otherwise symmetric waving flagellum (**Figure** **3**A–F), captured by the parameter *B* in Equation (1) below, and (b) a static curvature bias (Figure 3I–N), κ_0_ in Equation (2), that deforms the flagellum into a static curved shape from which symmetric beating is overlaid, see details below. For simplicity, the two waveform models are denoted as “*xyz*‐model” and “κ‐model,” respectively.

Below, we show the waveform models that dictate the 3D beating patterns at the body frame coordinates ξ, and are provided in Equations (1) and (2). The *xyz*‐model employs the one‐sided bias beating asymmetry (Figure 3A–F). Methods Section and Table 2 describe the full list of definitions in this work. The waveform ξ‐coordinates are prescribed directly at the body frame of reference,

(1) $$\begin{array}{cc} \xi_{1} & =A\left(\xi_{3}\right)\left[\cos(k\xi_{3}-t)+B\right]\\ \xi_{2} & =-\alpha \;A\left(\xi_{3}\right)\sin\left(k\xi_{3}-t\right), \end{array}$$

where *A*(ξ_3_) = 0.2ξ_3_ is the modulating amplitude growing linearly with ξ_3_,^[^ ^66^, ^67^ ^]^ and *k* is the wave number, taken to be low (*k* = π, 2π, 3π) according to the estimations from the observed waving patterns in low viscosity.^[^ ^35^, ^57^, ^58^ ^]^ The maximal value of ξ_3_ coordinate is not specified here, and is instead subject to the constraint of the normalized arclength of the flagellum (see Methods Section), given by $s=\int_{0}^{\xi_{3}}\sqrt{1+{\left(d\xi_{1}/d\xi_{3}\right)}^{2}+{\left(d\xi_{2}/d\xi_{3}\right)}^{2}}d\xi_{3}$, with the total arclength equal to one. The flagellar shape is defined by coordinates (ξ_1_, ξ_2_, ξ_3_), and thus ξ_3_ coordinate does not represent a constant material point along the flagellum. *B* introduces the static one‐sided shifting asymmetry, and α captures the out‐of‐plane motion of the beat, responsible for the rotation amplitude of the sperm flagellum in the body frame. If *B* = 0, the waveform is perfectly symmetric (Figure 3A,F), otherwise it yields an average flagellum shifted sideways relative to the head long axis $\xi_{3}$ red line on the ξ_1_ξ_3_ plane in Figure 3B–E). If α = 0, the waveform is planar (Figure 3A,B), whilst when α increases, the flagellum follows an elliptical path in the cross section, with perfect circular trajectories when α = 1, see the projected point clouds on the ξ_1_ξ_2_ plane in Figure 3C–F. The sign of α dictates the chirality of flagellar beat, with a positive sign inducing a left‐handed helicoid (Movie S8, Supporting Information) and a negative sign inducing a right‐handed helicoid (Movie S9, Supporting Information). The rotational direction of the head spinning around its longitudinal axis $\xi_{3}$ is opposite to that of the tail due to the torque balance,^[^ ^54^ ^]^ see Movies S8 and S9 (Supporting Information). Numerical simulations using *k* = 2π are provided in the main text, and *k* = π, 3π are supplied in Supporting Information.

The second type of waveform asymmetry due to a curvature bias is introduced via the κ‐model (Figure 3I–N), in which waveform curvature κ and torsion τ are prescribed instead,

(2) $$\begin{array}{c} \kappa =\kappa_{0}+\cos\left(ks-t\right). \end{array}$$

κ_0_ represents the static curvature over one beat cycle. According to the experimental measurements,^[^ ^6^, ^26^, ^36^, ^38^ ^]^ κ_0_ and τ are held as constants, ranging from 0 to 0.6 and from 0 to 5, respectively. If κ_0_ = 0, the waveform is symmetric (Figure 3I,L, and M), while a non‐zero κ_0_ gives rise to a curved average shape of the flagellum (Figure 3J,K, and N). The out‐of‐plane motion of the flagellum is controlled by τ, with τ = 0 generating a planar waveform (Figure 3I,J), and a larger τ producing a larger rotation amplitude of the flagellum at the body frame, with increasing circular motion with increasing τ (Figure 3K–N). For comparison purpose, the wave number *k* for the κ‐model was set to 2π. With specified curvature and torsion, the waveform at the body frame is obtained by integrating the local Frenet–Serret system of equations,

(3) $$\begin{array}{c} \frac{d\xi }{ds}=T,\frac{dT}{ds}=\kappa N,\frac{dN}{ds}=-\kappa T+\tau B,\frac{dB}{ds}=-\tau N. \end{array}$$

where *d*/*ds* is the derivative with respect to arclength, and T,N,B represent the tangent, normal, and binormal unit vectors of the local Frenet–Serret frame, from which the body frame coordinates ξ of the prescribed flagellum, used in Equation (2), are obtained.

Once the sperm's flagellar waveform is set in the body frame (using the models described above), we can calculate the swimming motion of the sperm in the lab frame by ensuring that hydrodynamic forces and torques are balanced (see Methods Section). To understand how the waveform affects sperm swimming in the lab frame (Figure 1B), we measure two key motion types: (i) the path of the sperm's head as it moves forward (shown by the blue curve in Figure 1C), and (ii) the 3D rotation of the sperm's head as it swims (depicted by the red, green, and blue trajectory lines in Figure 1B). To visualize the head's orientation, we map its motion onto a sphere in a moving frame of reference that travels with the sperm–this is called the orientation orbit (Figure 1D). The sperm's swimming path in the lab frame forms a periodic shape that matches the flagellar beat cycle, with the overall direction indicated by the red dotted line (Figure 1C). For clarity, all swimming paths are aligned to this forward direction (see Methods Section). In the comoving frame (Table 2), the orientation of the sperm's head traces three ring‐like paths on the sphere (Figure 1D). The axis passing through the center of these rings defines the head's precession axis (black line in Figure 1D). Importantly, the tilt of these orientation orbits with respect to the precession axis reveals crucial details about the symmetry of the flagellar waveform. This is key for detecting asymmetry, as it cannot be determined by looking at the swimming path alone (Figure 1C).

### 2.2 Virtual Swimming Paths Recapitulate the Diversity of Experimental Sperm Trajectories in 3D

We start by looking at how sperm swim in the lab frame, focusing on the variety of swimming paths generated by different flagellar waveforms. Figure 2 and 3 show the range of swimming patterns that emerge from varying two key factors: the asymmetry in the flagellar beat (*B*, κ_0_) and the out‐of‐plane flagellar amplitude (α, τ), as defined in Equations (1)–(3). Based on their shape, these swimming paths are grouped into six types: straight ribbon (SR), circular ribbon (CR), helical ribbon (HR), twisted ribbon (TR), spinning star (SS), and helical loop (HL), following Daloglu et al.^[^ ^44^ ^]^ (Figure 2). SR and CR are produced by planar waveforms and create “ribbon‐like” paths (Figure 2A,B and Figure 3A,B and I,J). SR traces are straight progressive paths, while CR traces are curved and non‐progressive. To characterize the path type and whether the sperm is moving in a flat plane or in 3D space, we measure the cell's linear velocity ($u_{\xi_{2}}$) along the direction perpendicular to the flatten side of sperm head (head basis vector $\xi_{2}$ in Figure 1B). For SR and CR, this velocity stays at zero, meaning the sperm is swimming in a flat plane (Figure 2A,B and Figure 3A,B and I,J). In contrast, the HR and TR patterns fluctuate slightly, though sufficiently to generate large 3D motion (Figure 2C,D and Figure 3C,D and K,L). Their velocity fluctuation stays below 1.5 × 10^−3^ (in dimensionless units). However, SS and HL patterns display pronounced 3D complex movement (Figure 2E,F and Figure 3E,F and M,N), with larger velocity fluctuations, as detailed in Methods Section. Finally, looking at the global shape of the HR, TR, SS, and HL paths (shown by magenta line in Figure 3C–F and K–N), we see they all form helical shapes. HR has a larger helix radius than TR. SS paths have sharp, star‐like corners, while HL paths show smaller periodic loops distributed around the helical ring (also in Figure 1C). Interestingly, aside from the planar CR path (Figure 2B and 3B and J), which is non‐progressive when the flagellar beat is asymmetric, all non‐planar waveforms, including quasi‐planar ones, result in progressive swimming, regardless of the symmetry state or waveform model used, even when the beat patterns are highly asymmetric, and for both types of asymmetry (waveform shift *B* or static curvature κ_0_) (Figure 2C–F and 3C–F and K–N).

Figure 2 and **Table** **1** show good agreements with representative experimental sperm trajectories,^[^ ^43^, ^44^ ^]^ with both *symmetric* and *asymmetric* waveforms, highlighting the validity of the framework employed, and in further agreement with early studies.^[^ ^6^, ^26^, ^36^, ^37^, ^38^, ^45^, ^54^, ^68^, ^69^ ^]^ Simpler trajectory patterns, such as SR, CR and HR, can only be obtained by either symmetric (SR) or asymmetric (CR, HR) waveforms, whereas Figure 2D–F and Figure S1D–F (Supporting Information) show that TR, SS, and HL modes are not exclusive to symmetric or asymmetric beating patterns. Asymmetric waveforms can generate almost indistinguishable swimming trajectories to the symmetric ones in 3D, thus both are able to provide good agreement with experiments at a qualitative level. Finally, in Table 1 we compare our numerical results for the HL mode with experimental measurements of sperm motion from Ref. [43], showing good accordance, further validating our simulations, waveform models and parameter choices quantitatively.

Figure 3 highlights how small differences in waveform provoke large distinctions in swimming paths. Fine structures of the paths are recorded in Movie S1 and S2 (Supporting Information), where intricate cusp formation and sharp turns are highlighted, as exemplified in Figure 3N. In particular, the trace patterns of HL mode exhibit local loops and global revolutions (Figure 1) with opposed chirality, similar to tracks previously reported in Refs. [44, 57]. As noted in the previous section, the simulated swimming paths displayed here are aligned to the *x*‐axis for comparison purpose (Figure 1). However, our simulations show that the resulting direction of progressive swimming is initial condition dependent and influenced by both waveform asymmetry (*B*, κ_0_) and the out‐of‐plane beat parameters (α, τ), see Movie S3 (Supporting Information). This suggests that sperm may adjust these factors–waveform asymmetry and the rotational component of the beat–to navigate in 3D environments with changing conditions.

Figure 3G,O, and Figure S2 (Supporting Information), show the diversity map of trajectory‐type in the asymmetry‐rotation parameter space (*B* − α and κ_0_ − τ). When the flagellar rotation amplitude is small (α, τ low), the waveform asymmetry dictates the variations of swimming patterns. On the other hand, when the out‐of‐plane component is high (α, τ high), the waveform asymmetry has negligible influence on the trajectory type; with the exception of HL for the κ‐model, which is only possible for large values of κ_0_ and τ in Figure 3O. For example, TR switches to HR by increasing *B*, for α between 10^−3^ and 10^−2^, whilst for α larger than 10^−2^, the trajectory‐type is independent of *B*, as similarly observed for the κ‐model.

Figure 3H,P, along with Figure S3 (Supporting Information), demonstrate how progressive swimming speed (**Table** **2**) changes based on two factors: waveform asymmetry (*B* or κ_0_) and the rotation amplitude (α or τ). As expected, symmetric waveforms produce the highest swimming speeds. While asymmetry generally slows down forward motion, the out‐of‐plane component (represented by α or τ) can help offset this reduction. In other words, increasing the rotational amplitude compensates for the negative effect of asymmetry on swimming speed. Interestingly, the effect of α and τ on both κ‐ and *xyz*‐models is non‐linear, meaning there's an optimal level of rotation amplitude that maximizes progressive speed for a given asymmetry.

**Table 2:** Key concepts and definitions.

Concept	Definition
Laboratory frame of reference (lab frame)	Fixed frame of reference where sperm swimming is observed.
Comoving frame of reference (comoving frame)	A frame of reference that translates but does not rotates with the sperm head.
Body frame of reference (body frame)	A frame of reference that translates and rotates with the sperm.
Waveform	Flagellar beating pattern relative to body frame.
Waveform symmetry state	Refers to whether the average flagellum shape is biased away from (asymmetric) or aligned with (symmetric) the sperm head's long axis, as shown by the red line projection on the ξ_1_ξ_3_ plane in Figure 3. In the *xyz*‐model, asymmetry is a side‐shift captured by *B*, with *B* = 0 representing symmetry. In the κ‐model, asymmetry is a static curvature controlled by κ_0_, with κ_0_ = 0 indicating symmetry.
Waveform rotation amplitude and the out‐of‐plane component of the beat	A waveform parameters: α and τ in the *xyz*‐model and κ‐model, respectively. When α = 0 (τ = 0), the waveform is planar. Otherwise, the flagellum follows an elliptical path in the cross section, as shown by the point cloud patterns of the end flagellar points in Figure 3.
Head center trajectory	The path traced by the sperm head center in the lab frame, capturing the translational movement of the sperm. It is characterized by sawtooth oscillations due to periodic flagellar beating (blue curve in Figure 1C).
Progressive swimming axis	The axis representing the global forward motion (and direction) of the sperm head center trajectory, shown as the red dotted line (red arrow) in Figure 1C.
Head orientation orbits	The orbits traced by the three head basis vectors $\xi_{1,2,3}$, representing the rotational motion of the sperm head (Figure 1D).
Head precession axis	The axis around which the three head orientation orbits revolve, represented by the black line in Figure 1D.
Progressive swimming speed	The linear speed of head center trajectory along its progressive swimming direction.
Trajectory pitch (*P*)	The longitudinal distance the sperm travels per revolution of its helical trajectory (Figure 5A).
Trajectory radius (*r*)	The radius of the transverse envelope of the helical trajectory revolution (Figure 5B).
Period of helical trajectory revolution (Δ*T*_*tra*_)	The time required for the sperm to complete one full cycle along its global helical or circular (applicable only for CR mode) path.
Head tilt angles ($\psi_{\xi_{1,2,3}}$)	The average angle between the head precession axis and the orientation orbits of the head basis vectors $\xi_{1,2,3}$ (Figure 5C–E).
Period of orientation orbit rotations (Δ*T*_*rot*_)	The time required for the sperm to complete one full rotation of its orientation orbit.

**Figure 4:**
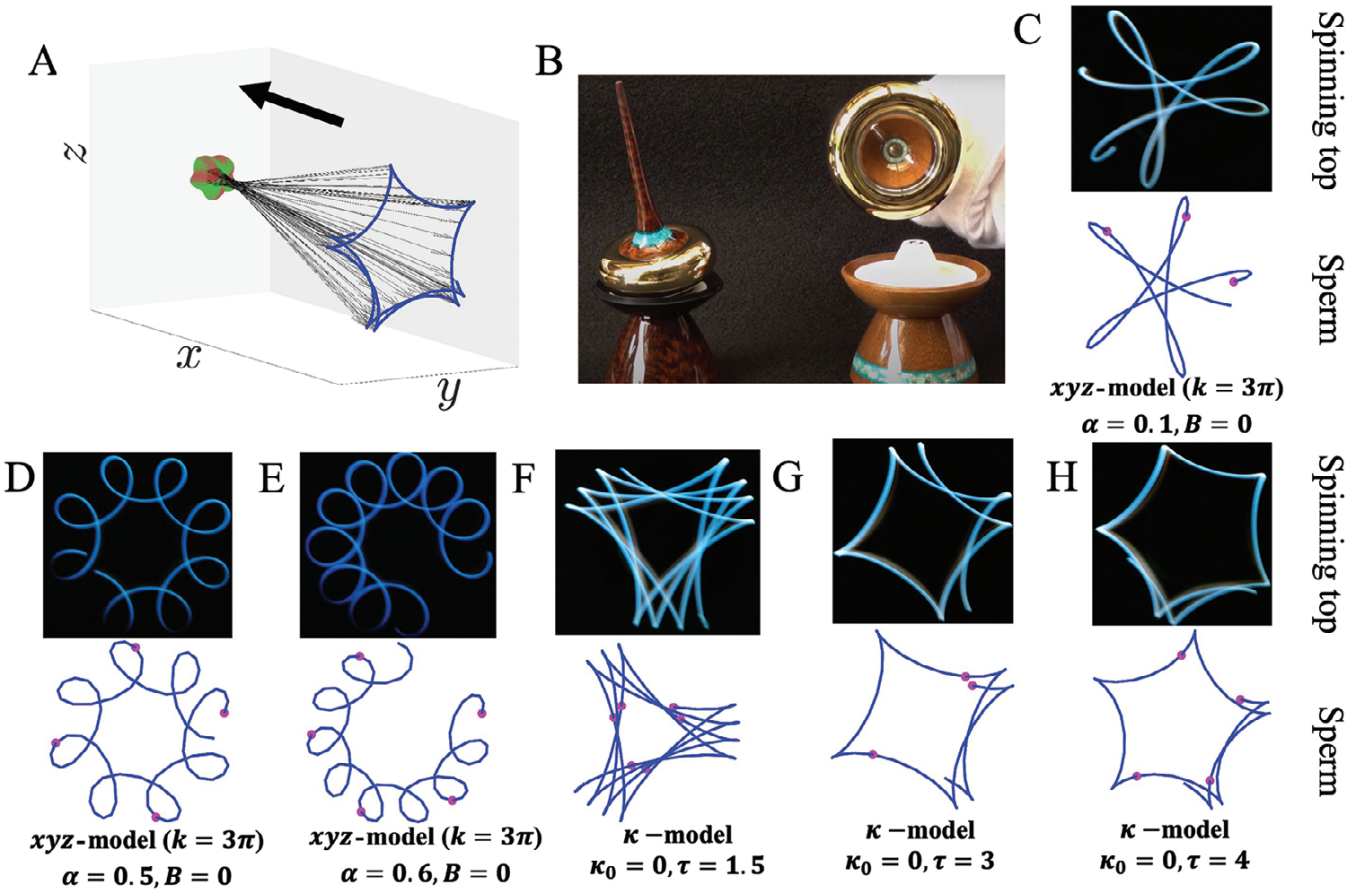
The trajectory of the spinning top's long axis closely resemble the sperm head's longitudinal axis, both displaying local loops or cusps around a global circle. A) Schematic illustrating the trajectory along the negative side of the sperm head's long axis $\xi_{3}$ (from the head center to the flagellum), with the sperm swimming direction indicated by a black arrow. The trajectory of the head's longitudinal axis, at a distance from the head center, forms a five‐point cusp pattern. B) Configuration of the observed ‘bottom‐heavy’ spinning‐top^[^ ^77^ ^]^ with its center of mass below the tip. Reproduced with permission.^[^ ^77^ ^]^ Copyright 2021, YouTube. The upper graph in each column of C–H) shows experimental images tracking the spinning top's long axis^[^ ^78^ ^]^ and the lower graph displays our simulated traces of the sperm head long axis in a similar configuration. The parameters of the virtual sperm models are listed at the bottom. Reproduced with permission.^[^ ^78^ ^]^ Copyright 2016, YouTube. Magenta points along the sperm trajectories are placed at intervals corresponding to the waveform beat cycle, indicating the synchrony between the flagellar beating and the helical path. In some κ‐model cases, the trajectory patterns shifts from loops to cusps as the distance along the head axis increases in A), see Figure S4 (Supporting Information).

We conclude this section by highlighting an interesting similarity between the trajectories of the sperm head's long axis and the motion of spinning tops.^[^ ^70^, ^71^, ^72^, ^73^ ^]^ **Figure** **4** compares the trajectory of a spinning top's long axis (Figure 4B) with the trajectory of the sperm head's longitudinal axis $\xi_{3}$ (Figure 4A) as it swims. Both show local loops (or cusps) that trace a global revolution around a central point. The spinning top in Figure 4B is a “bottom‐heavy” type, where its center of mass is below the spinning tip. This configuration causes loops to point outwards, with a mix of precession (slow, circular movement) and nutation (small wobbles). When nutation is minimal, the loops become cusps, as shown in Figure 4F–H. The trajectory of the sperm head's long axis follows a similar pattern in Figure 4C–H, with slight variations depending on the tracer distance from the rotation point. For some κ‐model cases, the trajectory changes from loops to cusps as the distance along the head axis increases (see Figure S4, Supporting Information). Figure 4 shows the trajectory of a point on the negative side of $\xi_{3}$, pointing from the head center to the flagellum (Figure 4A), which matches the configuration of the observed spinning‐top tracer in Figure 4B. In sperm swimming, the nutation‐like motion of the head, combined with the waveform's characteristics, creates these spinning‐top‐like patterns. It is important to note that the trajectory patterns in Figure 4 are for waveforms with large rotation amplitudes (α and τ), and larger values of these parameters lead to denser loops and cusps. Despite these visual similarities, however, the two systems differ fundamentally. The spinning top's motion is governed by rotational inertia,^[^ ^71^, ^74^, ^75^, ^76^ ^]^ while sperm motion occurs in an inertia‐less environment, with entirely different physics at play.

### 2.3 Waveform Asymmetry is Suppressed in the Sperm Swimming Paths but Manifested in the 3D Orientation Orbits

In this section, we explore the impact of waveform symmetry on progressive motion and head rotations in sperm swimming. This examination focuses on how sperm rotational dynamics are influenced by waveform characteristics, and how these rotations interplay with translational movements.

**Figure 5:**
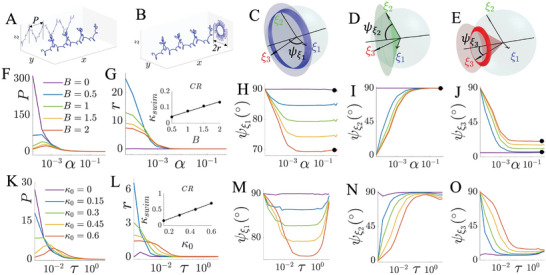
Parameterization of sperm locomotion, varying with waveform asymmetry and out‐of‐plane component. A) and B) Definitions of the longitudinal (*P*) and transverse (*r*) envelopes of aligned sperm head trajectory. C–E) Definitions of the head tilt angles $\psi_{\xi_{1,2,3}}$, the average angle between the head precession axis and the orientation orbits of $\xi_{1,2,3}$. F–J) Parameterization of the *xyz*‐model with *k* = 2π, including *P*, *r* and $\psi_{\xi_{1,2,3}}$. The inset of G) is for the CR mode with α = 0, and demonstrate a linear relationship between trajectory curvature κ_*swim*_, inverse of the average path radius, and waveform asymmetry. The dots in H–J) mark the 2 cases shown in Figure 7. K–O) Same results but for the κ‐model. Note that the zero origins in F–O) pertain to the vertical axis, and not the logarithmic horizontal axis representing α (τ)—zero is not displayed in horizontal axis.

The sperm head center position (path trajectory) revolves around the progressive swimming axis, while the sperm head rotates around the head precession axis (orientation orbits), as depicted in Figure 1C,D. Movie S4 (Supporting Information) shows that the change in head orientation is synchronized with the progressive swimming motion when adjusting the waveform parameter α. We define the angle between the progressive swimming axis and the head precession axis as γ. Figure S5 (Supporting Information) presents statistics of γ, where the medians and interquartile ranges vary closely around zero, with larger angles ranging between 10 and 30 degrees. This indicates that the trajectory's progressive axis is nearly aligned with the head precession axis, implying that the head basis vectors $\xi_{1,2,3}$ generally revolve around the progressive swimming axis as the head spins around $\xi_{3}$. **Figure** **5**J,O, and Figure S6E,J (Supporting Information), display the tendency of $\psi_{\xi_{3}}$ (described below) to decrease towards 0°, suggesting that a larger waveform rotation amplitude (α and τ) causes the head's long axis to move nearly parallel to both the head precession axis and the progressive swimming axis.

We measured the pitch and radius of the helical head trajectories (*P* and *r*), as well as the tilt angles of head orientation ($\psi_{\xi_{1,2,3}}$) in the lab frame, as defined in Figure 5A–E (parameters are displayed in Figure 5F–O and Figure S6, Supporting Information). Both *P* and *r* are affected by the waveform asymmetry, and at the same time, regulated by the out‐of‐plane component of the beat. As α and τ increase and the waveform becomes more circular in the cross‐section, the large pitch and radius in Figure 5F,G,K, and L drop, and ultimately decay to zero, regardless of the level of asymmetry. This indicates a transition from large helical paths (small α, τ) to a linear forward movement (large α, τ), see Movie S4 (Supporting Information). When the rotation amplitudes α and τ are zero, the Circular Ribbon (CR) mode trajectory displays a characteristic circular path (non‐progressive swimming). This path's curvature, κ_swim_, which is the inverse of the average path radius, exhibits a linear relationship with waveform asymmetry. This relationship is depicted in the insets of Figure 5G,L, and Figure S6B,G (Supporting Information), aligning with findings from Zaferani et al.^[^ ^37^ ^]^ and Friedrich et al.^[^ ^36^ ^]^ Figure 5F,G,K, and L shows that any distinction between the swimming paths due to the waveform asymmetry is lost as α and τ are increased (all curves collapse to zero). This indicates that the flagellum rotation amplitude suppresses the manifestation of waveform asymmetry at the swimming path level whilst promoting global progressive motion.

As described in Section 2.1, to track the orientation of the sperm's head, we project its motion onto a sphere in a frame of reference that moves with it, referred to as the orientation orbit (Figure 1D), whilst the axis passing through the center of these trajectories defines the head's precession axis (Figure 1D). Figure 5C–E, H–J, and M–O shows that the waveform asymmetry instigates complex rotational orbits in 3D. When α and τ are varied, the tilt angles of the sperm head orientation shown in Figure 5H–J and M–O remain fairly constant for symmetric waveforms, whilst those for asymmetric waveforms are affected noticeably— see comparisons provided in Movies S4 and S5 (Supporting Information), where the orbits of the head orientation vector $\xi_{2}$ are regularized, in contrast to the wobbling traces of $\xi_{1,3}$. As α (τ) increases, $\psi_{\xi_{2}}$ and $\psi_{\xi_{3}}$ tend to approach 90° and 0°, respectively, but asymptote to slightly different angles depending on the level of waveform asymmetry. The head orientation of asymmetric cases, for the basis vectors $\xi_{2,3}$, align more closely with the corresponding symmetric cases, as the flagellum rotation amplitude increases, indicating the suppression of rotation in two orientation directions as α and τ increase. Most importantly, the orientation orbits of $\xi_{1}$ differ dramatically depending on the level of waveform asymmetry, even when the out‐of‐plane component of the beat (α, τ) is large (Figure 5C,H,M; Figure S6C,H, Supporting Information). This indicates an asymmetry‐dependent effect on the dynamics of $\xi_{1}$, as well as $\xi_{3}$ for the *xyz*‐model with the shift bias *B* (Figure 5J; Figure S6E,J, Supporting Information). In the case of static asymmetric curvature κ_0_ in the κ‐model, Figure 5M, this distinction is most pronounced within the approximate α range of 10^−2^ − 10^0^, but becomes less significant for τ values greater than 2.

### 2.4 Waveform Rotation Inhibits Asymmetry in the Orientation Orbit Cycling Period

**Figure 6:**
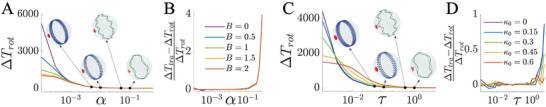
Periods of sperm cyclic movements, rotation of head orientation orbits (Δ*T*_*rot*_) and revolution of head center trajectory (Δ*T*_*tra*_), in function of waveform asymmetry and out‐of‐plane component. A) Orientation orbit periods of the *xyz*‐model using *k* = 2π. Insets exemplify the orientation orbits within one Δ*T*_*rot*_, with their spatial frequency decreasing when α increases. B) Relative deviation between the trajectory revolution cycle and the orientation rotation period, for the *xyz*‐model using *k* = 2π. C–D) Same results but for the κ‐model.

We continue our investigation of the impact of waveform symmetry on head rotations, now focusing on how asymmetry influences the timing of orientation orbit cycling periods. The time sperm takes to complete one period along its helical or circular path (Figure 1C) is defined as Δ*T*_*tra*_, and the time head basis vectors complete one orientation orbit (Figure 1D) is defined as Δ*T*_*rot*_, in units of the beat cycle. **Figure** **6**A,C, and Figure S7A,C (Supporting Information), show the orbital period of the head rotation, Δ*T*_*rot*_, as a function of α, τ for distinct waveform asymmetries (*B*, κ_0_). The smaller Δ*T*_*rot*_ period is, the faster the angular speed of head rotation will be. When α, τ are small, Δ*T*_*rot*_ can be as large as thousands of beat cycles, depending on the level of asymmetry. However, as α, τ increase, Δ*T*_*rot*_ decreases, regardless of the magnitude of the waveform asymmetry, with all cases collapsing into a fast sperm rotating mode, dominated by the waveform rotation amplitude. At larger values of α and τ, the sperm rotates much faster (as shown in Movies S6, S7, and S8, Supporting Information), but this fast rotation is characterized by a smoother wobble and a lower spatial frequency, as shown in the insets displaying one complete orbit. This suggests that larger waveform rotation amplitudes reduce the influence of waveform asymmetry on the timing of the sperm's orientation orbital cycles.

Figure 6B,D, and Figure S7B,D, (Supporting Information) illustrate the relative deviations between the head's helical trajectory period, Δ*T*_tra_, and the head's rotation orbital period, Δ*T*_rot_. For most of the studied range of α and τ, the deviation is either zero or very small, suggesting that the head's trajectory and rotational movements follow similar behaviors. For example, the decrease in Δ*T*_rot_ caused by increasing *B* (κ_0_) at small α (τ) implies both a shorter head rotation period and a faster revolution of the translation trajectory for a quasi‐planar waveform with more asymmetry. However, discrepancies between the translation and rotation periods occur when the waveform's out‐of‐plane component becomes very large. Movie S7 (Supporting Information) demonstrates sperm motion where the head's trajectory and rotation are in perfect sync, with zero deviation and α = 0.05. In contrast, Movie S8 (Supporting Information) shows a case where the head spins faster than its trajectory revolves, with a deviation of 3.2 beat cycles for α = 0.5.

### 2.5 Sperm Head Orientation in 3D is Essential for Detecting Waveform Symmetry

In the following sections, we evaluate how our results regarding sperm trajectory and head orientation impact two common methods used to detect flagellar symmetry in free‐swimming sperm. The first one employs 3D tracking of the sperm flagellum at the lab frame,^[^ ^44^, ^58^, ^61^, ^63^ ^]^ the second uses Computer‐Assisted Sperm Analysis (CASA) to infer motility parameters from sperm head trajectories in 2D, commonly used for semen analysis.^[^ ^59^, ^79^, ^80^ ^]^

**Figure 7:**
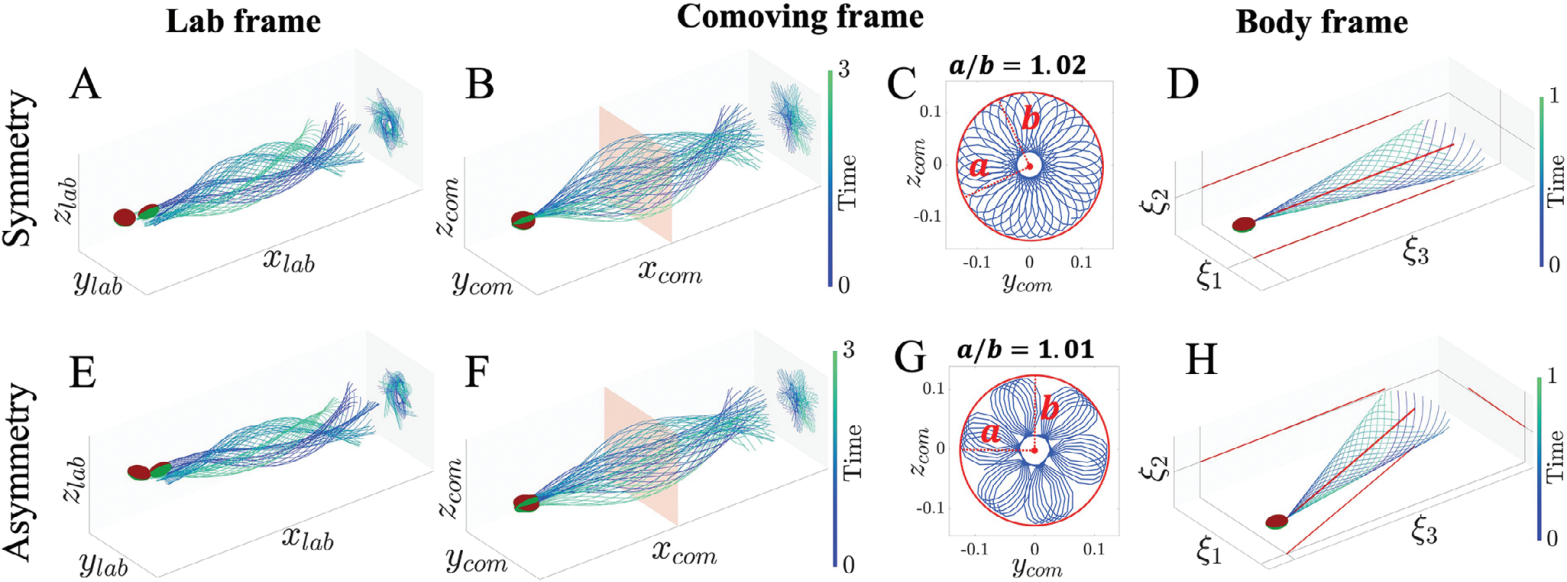
Flagellar beats in the laboratory, comoving and body frames of reference, where the top row is for a symmetric waveform model, and the bottom row is for an asymmetric case. The beatings in the lab frame, A) and E), and comoving frame, B) and F), are within one complete trajectory revolution period, which is three beat cycles for the cases examined. Flagellar projections are shown on the *yz* plane and, particularly, for the comoving results, red planes are used to indicate the approximate time‐varying positions of the mid‐flagellum points, whose projections are shown in C) and G), where red “ellipse” obtained via PCA captures the out‐of‐plane motion of the flagellum. The ellipse ratios of the major axis to the minor axis, *a*/*b*, equal to 1.02 and 1.01, respectively, for the symmetric and asymmetric cases. This indicates symmetric circular distribution of trajectories around the center. Body frame waveforms in D) and H) are symmetric and asymmetric, respectively, as indicated by the red average flagellum shape on the ξ_1_ξ_3_ plane, which is either aligned with or biased away from the $\xi_{3}$ head long axis.

As presented above, sperm rotation tends to “filter out” the effects of waveform asymmetry from the linear translation and swimming paths. **Figure** **7** further demonstrates this by showing flagellar beating patterns in the lab (A, E), comoving (B, F), and body frames of reference (D, H) for both symmetric and asymmetric waveforms. The flagellum performs 3D motion around the swimming direction (Figure 7B,F). Projections on the *yz*‐plane exhibit isotropic and symmetric distributions of waveform tracers around the *x*_com_ plane for both symmetric and asymmetric cases (Figure 7B,F). Principal component analysis (PCA) of mid‐flagellar tracers (Figure 7C,G) reveals that the semi‐axes ratio (*a*/*b*) of the fitted ellipses is close to one, indicating symmetry in all flagellar motions (Figure 7C,G). Thus, the waveform symmetry remains indistinguishable for both flagellar tracers at the lab and comoving frames. This can be however circumvented by directly tracking both sperm head orientation and flagellar waveform in 3D, to allow reconstruction of the true flagellar beat in the body frame of reference, where the symmetry state can be directly inferred. Figure 7D,H clearly shows the distinction between asymmetric and symmetric beating patterns in the body frame.

Waveform symmetry detection requires 3D head orientation information for free swimming cells. This is evident in Figure 5H–J, where angular differences between asymmetric and symmetric cases are clearly seen, though this is dependent on the type of asymmetry. Figure 5H,M, and Figure S6C,H (Supporting Information) show that 3D angular movements of the sperm head may be used to distinguish between asymmetric and symmetric waveforms, even for large values of α and τ. For symmetric cases (*B* = 0, κ_0_ = 0), the head orientation relative to the precession axis, quantified by $\psi_{\xi_{1,2,3}}$, remains unchanged with varying α or τ. However, this changes with the type of waveform asymmetry (compare Figure 5H,M, for example). So, in principle, the 3D orientations in Figure 5 could be used as a lookup table to infer waveform asymmetry. For symmetric cases, $\psi_{\xi_{1}}=\psi_{\xi_{2}}=90^{\circ }$, and $\psi_{\xi_{3}}$ values are approximately 7°, 5.5°, 3.6°, and 6.6° for the *xyz*‐model with *k* = π, 2π, 3π, and the κ‐model. Movie S4 (Supporting Information) illustrates how the orientation orbits of asymmetric waveforms vary with α, in comparison to symmetric cases shown in Movie S5 (Supporting Information).

**Figure 8:**
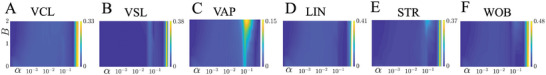
Kymographs of the relative deviations between 2D and 3D CASA parameters, based on the *xyz*‐model using *k* = 2π and varying with α and *B*. From A) to F): curvilinear velocity (VCL), straight‐line velocity (VSL), average‐path velocity (VAP), linearity (LIN, equal to VSL/VCL), straightness (STR, equal to VSL/VAP) and wobble (WOB, equal to VAP/VCL).^[^ ^59^ ^]^

CASA systems are widely used in clinical settings to assess sperm motility based on 2D tracers.^[^ ^80^, ^81^ ^]^ We present evaluations of generalized 3D CASA parameters based on our numerical 3D sperm trajectories, including curvilinear velocity (VCL), straight‐line velocity (VSL), average‐path velocity (VAP), linearity (LIN = VSL/VCL), wobble (WOB = VAP/VCL), and straightness (STR = VSL/VAP).^[^ ^59^ ^]^ Figure S8 (Supporting Information) and **Figure** **8** show the 3D CASA parameters and the deviation between 2D projections of 3D sperm movement as a function of waveform asymmetry and the flagellar rotation amplitude. The effect of waveform asymmetry is nearly indistinguishable in Figure S8 (Supporting Information) and Figure 8. CASA parameters are only weakly affected by waveform asymmetry (*B*) due to persistently progressive swimming motion in 3D (Figures 3, 5 and 7). Although 2D CASA parameters show only weak deviations from their 3D counterparts for most of the parameter space in Figure 8, the relative deviation can be as high as 33.21%, 38.68%, 15.69%, 41.26%, 37.94%, and 48.40% for VCL, VSL, VAP, LIN, WOB, and STR, respectively. The absolute difference between 2D and 3D for VCL can reach up to 28.71µm/s, which exceeds the threshold for distinguishing slow from rapid progressive motility (25µm/s), according to WHO guidelines,^[^ ^82^ ^]^ and constitutes approximately 1/5 of the speed threshold for hyperactivated motility (150µm).^[^ ^83^ ^]^ Thus, 2D measurements of 3D sperm trajectories may introduce inaccuracies in sperm motility analysis using CASA parameters.

## 3 Discussion

We have conducted numerical simulations of sperm swimming hydrodynamics to elucidate the role of intrinsic waveform asymmetry on the resulting 3D swimming movements. Numerical simulations were verified against experimental swimming observations and measurements,^[^ ^36^, ^37^, ^38^, ^43^, ^44^, ^68^ ^]^ with waveform model parameters estimated directly from experiments.^[^ ^24^, ^35^, ^38^, ^57^, ^58^, ^60^, ^63^, ^84^ ^]^ Our study reveals that waveform asymmetry may not appear in the swimming path patterns but instead affects the 3D head rotations in a complex way. Both symmetrical and asymmetrical beatings result in persistently progressive swimming, meaning that 3D sperm trajectories alone cannot determine the symmetry state of the beating pattern.

The sperm flagellum contains a variety of inherently asymmetric components, ranging from molecular to micron levels, such as molecular motors, radial spokes, elastic linkers, microtubules, outer dense fibers, centrioles, basal components, and ion channels.^[^ ^27^, ^28^, ^29^, ^30^, ^31^, ^32^, ^33^, ^34^, ^35^ ^]^ But how does sperm manage to swim forward with such an intrinsically asymmetric flagellar apparatus? Our simulations show that forward swimming is not impeded by waveform asymmetry, thanks to the regularizing effect of the flagellum's rotational motion. This enables the flagellar apparatus to drive cells forward despite any “imperfections” in the symmetry of the beat. The rotational motion of the sperm flagellum thus acts as a reliable mechanism for forward propulsion in nature, likely playing a critical role during the evolution of these structures to maintain biological function. Achieving a perfectly symmetrical flagellar apparatus with perfectly symmetric molecular to micron components would be nearly impossible. Our results suggest that asymmetries in the flagellar beat do not significantly affect the ability of sperm to swim forward, progressively for 3D beating patterns.

The sperm's ability to persistently swim in a progressive helical path, despite waveform asymmetry, does not hinder its capacity to steer and navigate in 3D. Varying levels of waveform asymmetry, modulated by flagellar rotation, lead to progressive paths in different directions (Movie S3, Supporting Information). This suggests that sperm can control navigation in 3D by adjusting waveform asymmetry and the flagellum rotation amplitude, without the risk of being “trapped” in circular swimming paths, as seen with asymmetric planar waveforms.^[^ ^18^, ^36^, ^37^, ^84^ ^]^ Waveform asymmetry increases the diversity of progressive swimming paths (Figure 3G,O; Figure S2, Supporting Information), and sperm rotations help avoid negative impacts on swimming speed (Figure 3H,P; Figure S3, Supporting Information).

Comparison between simulations and experimental sperm trajectories showed that both symmetric and asymmetric waveform models can qualitatively replicate observed patterns (Figure 2D–F). This means waveform symmetry cannot be determined solely from swimming trajectories, but also, comparing models with experiments based on trajectory data alone may be insufficient. To accurately compare free‐swimming sperm experiments with model predictions, head center trajectories should be analyzed alongside 3D head orientation dynamics, as performed in Ref. [85]. Given that past comparisons have primarily focused on head trajectories, this suggests an opportunity to reconsider model comparisons with experiments in future studies accounting for sperm rotations in 3D.

We have shown that 3D detection of flagellar beating in the lab frame may not be enough to detect beat asymmetry (Figure 7). Inferring asymmetry requires combining flagellar tracers in 3D with head rotations, as this enables the reconstruction of the “true” flagellar beat relative to the body frame of reference–viewed from a fixed point at the sperm head that moves with the cell in 3D (Figure 1A). Although the flagellum shape is the same in both lab and body frames, their position and orientation differ significantly. Body frame translations and rotations, for a given waveform, can be determined by solving force and torque balance equations (Methods Section), known as the mobility problem in low Reynolds number hydrodynamics.^[^ ^86^ ^]^ However, finding body frame information from the lab frame beating is not a well‐posed problem, as the flagellum centreline lacks orientation/rotational information of the body motion.^[^ ^85^ ^]^ Thus, 3D flagellar tracking without body orientation detection may not fully capture the complete picture of how the flagellum moves in the body frame. Indirect methods have been used to infer body orientation from 3D flagellar paths,^[^ ^56^, ^67^ ^]^ but further research is needed to better understand their effectiveness and accuracy. We hope these findings encourage further development of direct empirical methods for measuring 3D body orientation at microscale, such as in Refs. [85, 87].

In experimental and clinical studies assessing sperm motility, 2D measurements of 3D movements may oversimplify the true swimming motion. Figures 8 and Figure S6 (Supporting Information) show that CASA parameters are unable to differentiate between symmetric and asymmetric waveforms for sperm swimming in 3D, leading to potential errors when tracking 2D projections of 3D movements. This may be significant as current CASA systems are limited to 2D visualizations of sperm trajectories.^[^ ^23^, ^37^, ^87^, ^88^, ^89^ ^]^

Our numerical study on waveform asymmetry in 3D sperm swimming has several limitations. We focused on two types of empirically observed asymmetries–one‐sided waveform shifts and static curvatures–while other forms,^[^ ^6^, ^18^, ^24^, ^35^, ^38^, ^63^, ^90^ ^]^ such as second harmonics^[^ ^90^ ^]^ or inclined planar beating,^[^ ^18^, ^54^ ^]^ may also exist. Additionally, we only considered the fundamental mode of mammalian beating type observed in low viscosity fluids^[^ ^62^, ^84^, ^91^, ^92^ ^]^ and did not account for interactions with boundaries, which are known to influence sperm behavior near walls.^[^ ^18^, ^54^, ^64^ ^]^ Furthermore, we used prescribed waveform models, meaning the flagellar shape did not emerge spontaneously from molecular motor behavior.^[^ ^93^, ^94^ ^]^ Despite these simplifications, our results highlight the fundamental role of waveform asymmetry, the complex 3D rotational motion of sperm, and the diversity of persistently progressive swimming patterns. We hope this work will inspire future research into the role of asymmetry in cell motility, rotational motion of microorganisms, 3D waveform tracking, and artificial swimmers.

## 4 Methods Section

### 4.1 Numerical Simulations

A meshfree approach is exploited using the Regularized Stokeslet method (RSM) by Cortez–Fauci–Medovikov^[^ ^95^ ^]^ to solve the non‐local low Reynolds number hydrodynamics of sperm swimming. The RSM has been extensively studied and validated in the literature,^[^ ^53^, ^95^, ^96^, ^97^, ^98^, ^99^ ^]^ and the novel nearest‐neighbor discretization method developed by Gallagher–Smith^[^ ^86^, ^100^, ^101^ ^]^ was used in this work for efficient computations of the non‐local flow fields. Gallagher–Smith method offers model simplicity and versatility, and has been optimized and validated for free‐swimming problems, more details can be found in Ref. [86], including a didactic Matlab implementation of the method. By invoking total force and torque balance, this framework provides the free‐swimming motion of a spermatozoon, relative to the laboratory fixed frame of reference (lab frame), by prescribing the beating pattern of the flagellum relative to the body fixed frame of reference (body frame), i.e., the reference frame that translates and rotates with the sperm head (Figure 1). The microscale flow velocity at a spatial point x, driven by a regularized force $\phi^{\varepsilon }\left(x-X\right)f$ at the location X, can be represented as $u=G^{\varepsilon }\left(x,X\right)\cdot f$, where $\phi^{\varepsilon }\left(x-X\right)=\left(15\varepsilon^{4}\right)/\left[8\pi {\left(r^{2}+\varepsilon^{2}\right)}^{7/2}\right]$ is the cutoff function, and $G^{\varepsilon }=\left[\left(r^{2}+2\varepsilon^{2}\right)I+rr\right]/\left(8\pi r_{\varepsilon }^{3}\right)$ is the regularized Stokeslet, with r=x−X, r=|r|, ϵ is the regularization parameter, and $r_{\varepsilon }=\sqrt{r^{2}+\varepsilon^{2}}$.^[^ ^95^, ^97^ ^]^ The laboratory frame coordinates of the sperm are described as $x=x_{0}+R\cdot \xi$, where $x_{0}$ is the origin of the body frame, i.e., head center, $R=[\xi_{1},\xi_{2},\xi_{3}]$ is director basis capturing the orientation of the body frame, and ξ the body frame coordinates of the flagellum shape (Figure 3). The sperm velocity satisfies a surface integral over the body surface ∂*D*, for all x∈∂D,

(4) $$\begin{array}{c} U+\Omega \times \left(x-x_{0}\right)+R\cdot \dot{\xi }=\;\int {\int \;}_{X\in \partial D}G^{\varepsilon }\left(x,X\right)\cdot f\left(X\right)dS_{X}, \end{array}$$

where U and Ω are the unknown linear and angular velocities of the body frame, respectively, f is the unknown surface traction, and the overdot of ξ denotes a time derivative of the body frame coordinates. The above equation embodies the non‐local force‐velocity relationship and non‐slip boundary condition, which is augmented by the total balance forces and torques on the sperm,

(5) $$\begin{array}{ccc} & & \int {\int \;}_{X\in \partial D}f\left(X\right)dS_{X}=0\\ & & \int {\int \;}_{X\in \partial D}X\times f\left(X\right)dS_{X}=0. \end{array}$$

The above system of equations governs the so‐called mobility problem,^[^ ^86^ ^]^ in which the unknown rigid‐body motion results from imposed force and moment. In other words, the unknown traction f, and the translational U and rotational Ω velocities of the body frame can be obtained numerically from a prescribed waveform model relative to the body frame of reference. This allows us to resolve the translating and rotating 3D kinematics of the swimming sperm at the lab frame, examples of which can be seen in Figures 1 and 3. The system is treated as an initial‐value problem and solved via the built‐in function ode45 in MATLAB, and the algorithm is implemented as dimensionless, where the flagellum length is normalized to 1 and time is quantified in terms of beat cycles. Here, sperm swimming is considered in an infinite fluid and boundary effects were neglected for simplicity, though this could be easily incorporated^[^ ^64^, ^86^ ^]^ in future studies. The human sperm head has a marginal impact on sperm motility due to their typical small sizes (when compered against the flagellum length),^[^ ^4^, ^54^, ^64^, ^84^ ^]^ and thus the head geometry adopted here is simplified to a scalene ellipsoid with axes of length 0.044, 0.036, and 0.022.^[^ ^86^ ^]^ A finer quadrature discretization with 700 points was used for the sperm head (though this could be lower), and a coarser force discretization with 136 points was used for the flagellum, with the regularization parameter ϵ = 0.25/45 chosen to approximately represent the ratio of flagellar radius to length of human sperm [*ibid*]. Simulations were conducted on the High Performance Computing system of University of Bristol: BlueCrystal Phase 4.

### 4.2 Definitions and Quantification of 3D Sperm Swimming Motion

The reader can refer to Table 2 for descriptions of important concepts and definitions appearing in this work.

**Lab frame trajectory of sperm head**: The typical lab frame trajectory of the sperm head center, represented by the blue curve in Figure 1C, shows sawtooth‐like oscillations resulting from periodic flagellar beating. To remove local path oscillations and highlight the global helical structure of the trajectory, smooth spline fitting is applied to calculate the average path (green curve in Figure 1C), which minimizes the second derivative of the target curve and the distance from the original trajectory using least‐squares approximation.

**Progressive swimming axis and sperm head trajectory alignment**: The cyclic revolution of the helical average path determines the size of the sliding window used to calculate the progressive axis, shown by the red dotted line in Figure 1C. To facilitate the comparison and quantification across different trajectory types, this axis is used to align the sperm head trajectory, ensuring it runs parallel to the *x*‐axis. For the circular ribbon (CR) mode (definition below), which has no progressive motion, the trajectory is aligned by centering the average path at the origin.

**Sperm head orientation and rotation**: 3D orientation of sperm head in the lab frame of reference is indicated by the traces of three head basis vectors $\xi_{1,2,3}$ (Figure 1B), from which the head rotational movement is extracted to form the three orientation orbits in Figure 1D (one for each head basis vector). The lab frame trace of the head long axis ($\xi_{3}$) show patterns strikingly similar to the orbits of a spinning top's rotational axis (Figure 4), revealing spinning‐top type rotations of sperm swimming (Section 2.2).

**Head precession axis and orientation orbit alignment**: The centers of the three head orientation orbits form a straight line that defines the head precession axis (black line in Figure 1D), which nearly overlaps with the progressive swimming axis (Section 2.3). As sperm rotates around its head longitudinal axis $\xi_{3}$, the $\xi_{3}$ vector also precesses about the head precession axis (black line in Figure 1D), which is further aligned to be parallel to the *x*‐axis for easy comparison purpose.

**Classification of 3D sperm head trajectory patterns**: The diversity of head center trajectory (Figures 2 and 3) is linked to the linear velocity of the sperm head relative to its basis vector $u_{\xi_{2}}$, and as such, the velocity value and range (in dimensionless units) were used to quantitatively classify the trajectory patterns. With the known head orientation, $R=[\xi_{1},\xi_{2},\xi_{3}]$, and head center position, $x_{0}$, in the lab frame, the linear velocity was calculated using $u=R^{T}{\dot{x_{0}}}^{T}$, with the overdot representing time derivative. The six trajectory types are:

Straight ribbon (SR): planar trajectory mode corresponding to $u_{\xi_{2}}=0$, with its global trace moving forward in a straight line.

Circular ribbon (CR): planar trajectory mode corresponding to $u_{\xi_{2}}=0$, with its global shape following non‐progressive circular paths.

Helical ribbon (HR): quasi‐planar trajectory pattern with the oscillation range of its periodic velocity $u_{\xi_{2}}$ below 1.5 × 10^−3^. The global shape of HR mode forms a helix and its cross‐section demonstrates a relatively large helix radius.

Twisted ribbon (TR): quasi‐planar trajectory pattern with the oscillation range of its periodic velocity $u_{\xi_{2}}$ below 1.5 × 10^−3^. The global shape of TR mode forms a helix and its cross‐section demonstrates a relatively small helix radius.

Spinning star (SS): trajectory mode exhibiting pronounced 3D local features, corresponding to an oscillating range of $u_{\xi_{2}}$ above 1.5 × 10^−3^. SS path is helical in shape globally, with star‐like corners featuring its transverse pattern.

Helical loop (HL): trajectory mode exhibiting pronounced 3D local features, corresponding to an oscillating range of $u_{\xi_{2}}$ above 1.5 × 10^−3^. HL path is helical in shape globally, with its transverse pattern featured by periodic loops distributed around the helical ring.

**Quantification of sperm head translation**: Sperm linear movement (head trajectory) is aligned with the progressive axis by adjusting the latter to be parallel to the *x*‐axis (Figure 1C), and then quantified spatially and temporally through progressive swimming speed (Section 2.2), trajectory pitch and radius (Section 2.3), and period of helical trajectory revolution (Section 2.4).

Progressive swimming speed (Figure 3H,P: Figure S3, Supporting Information): linear speed of the head center trajectory in the lab frame along the progressive swimming direction (red arrow in Figure 1C).

Trajectory pitch (*P*, Figure 5A,F,K; Figure S6A,F, Supporting Information): the average longitudinal distance traveled per helical trajectory revolution.

Trajectory radius (*r*, Figure 5B,G,L; Figure S6B,G, Supporting Information): radius of the transverse envelope of the helical trajectory revolution.

Period of helical trajectory revolution (Δ*T*_*tra*_, Figure 6B,D, Figure S7B,D, Supporting Information): the time sperm takes to complete one period along its global helical or circular (only for CR mode) path.

**Quantification of sperm head rotation**: Sperm angular movement (head orientation) is captured by aligning the head precession axis with the *x*‐axis (Figure 1D), and then quantified through head tilt angles (Section 2.3) and period of orbit rotations (Section 2.4).

Head tilt angles ($\psi_{\xi_{1,2,3}}$, Figure 5C–E, H–J, M–O; Figure S6C–E,H–J, Supporting Information): the average angle between the head precession axis and the orientation orbits of head basis vectors $\xi_{1,2,3}$.

Period of orientation orbit rotations (Δ*T*_*rot*_, Figure 6A,C; Figure S7A,C, Supporting Information): the time sperm takes to complete one orientation orbit.

## Conflict of Interest

The authors declare no conflict of interest.

## Supporting information

Supporting Information

Supplemental Movie 1

Supplemental Movie 2

Supplemental Movie 3

Supplemental Movie 4

Supplemental Movie 5

Supplemental Movie 6

Supplemental Movie 7

Supplemental Movie 8

Supplemental Movie 9

## Data Availability

Data sharing is not applicable to this article as no new data were created or analyzed in this study.
