# Supplementary material for: Swimming by Spinning: Spinning‐Top Type Rotations Regularize Sperm Swimming Into Persistently Progressive Paths in 3D
Source: Adv Sci (Weinh). 2024 Dec 18;12(6):2406143. doi: 10.1002/advs.202406143 (PMC11809349; doi:10.1002/advs.202406143)
Supplement: Supplementary file 1 — Supporting Information [file ADVS-12-2406143-s008.docx]

Supporting Information

Swimming by spinning: spinning-top type rotations regularize sperm swimming into persistently progressive paths in 3D

Xiaomeng Ren and Hermes Bloomfield-Gadêlha*

**This PDF file includes:**

Supplementary Text

Figure S1 to S8

Movie S1 to S9

**Other Supplementary Materials for this manuscript include the following:**

Movies S1 to S9

Supplementary Text

Principal component analysis on comoving flagelloid

We analyze the flagelloid curves of the mid-flagellar points in the comoving frame of reference (Figure 7 C and G, in the main text) using principal component analysis method. The projected flagellar data on the YZ plane is written as a *n*
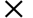
*2* matrix ***D***=[*Y(*
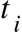
*), Z(*
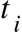
*)*], with time discretized into *n* values,
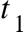
, …,
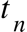
. Then we have the covariance matrix ***C***= (1/*n*)
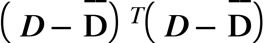
, where
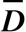
 is the temporal average result. The eigenvectors of ***C*** decide the semi-axes directions of the ellipse shown in Figure 7 C and G (main text), and the ratio between the semi-axes, *a/b*, is calculated using the associated eigenvalues.


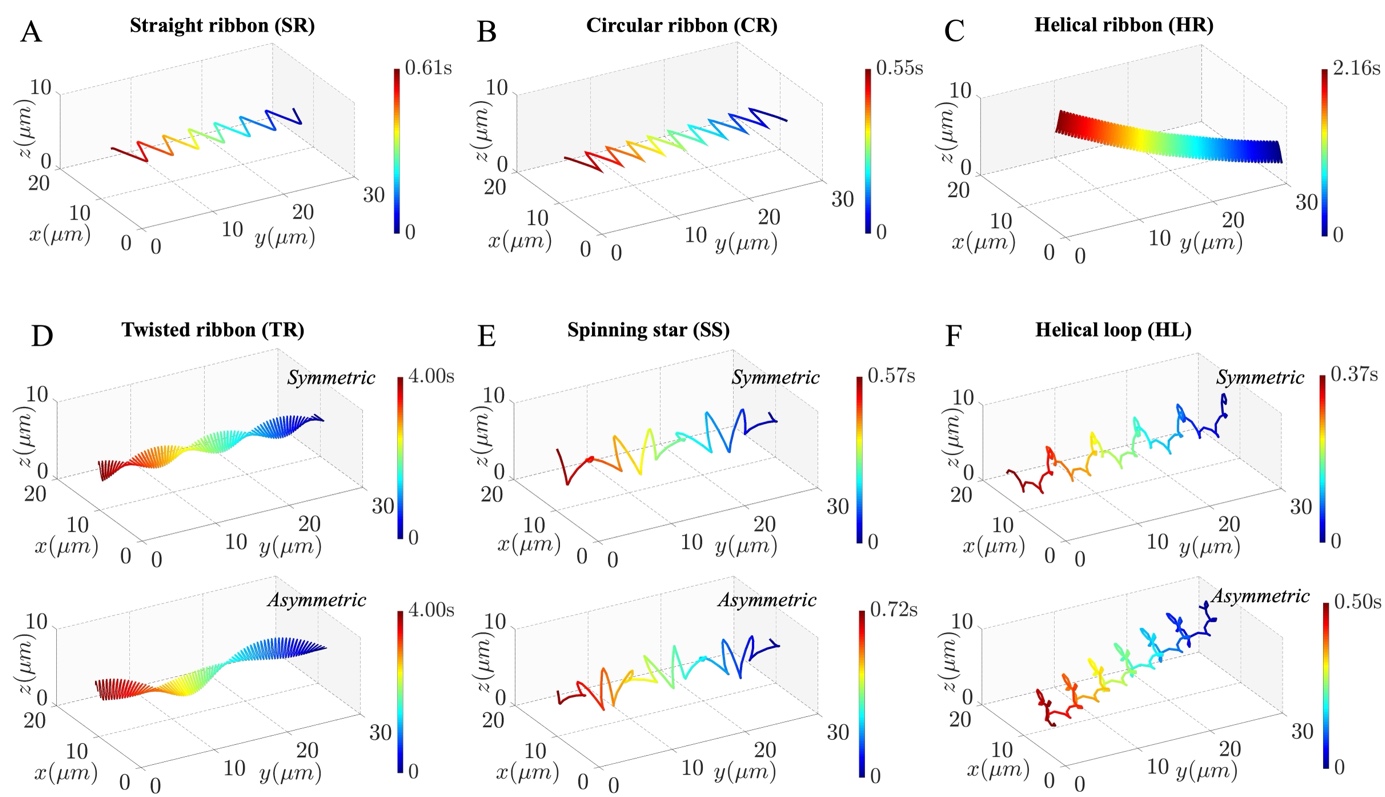


Figure S1.

Head center trajectories of virtual sperm models in the lab frame. The non-dimensionalized simulations have been scaled according to the bovine sperm arc length of 65$\mu m$ [1]-[5] and the flagellar beating frequencies from [1]. A)-F) depict the following trajectory modes: straight ribbon (SR), circular ribbon (CR), helical ribbon (HR), twisted ribbon (TR), spinning star (SS), and helical loop (HL), respectively. Time progression is represented by color gradients. To facilitate comparison, the viewing angles and scale ranges of all traces have been unified, highlighting the unique characteristics of each mode. Notably, the SR, CR, and HR modes can only be generated by either symmetric or asymmetric virtual models, while other modes can be reproduced by both.


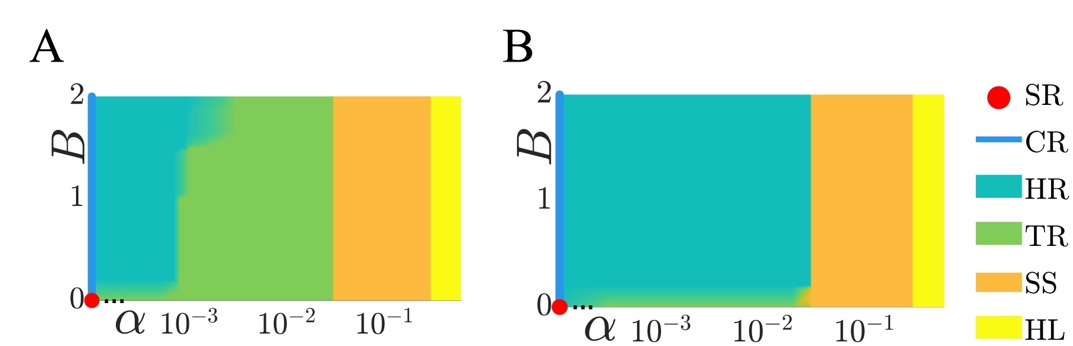


Figure S2.

Trajectory classification varies with waveform asymmetry (*B*) and rotation amplitude (
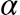
). The numerical trajectory results are classified into 6 modes: straight ribbon (SR), circular ribbon (CR), helical ribbon (HR), twisted ribbon (TR), spinning star (SS) and helical loop (HL).

The trajectory pattern transitions for the
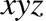
-model using
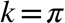
 and
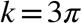
 are shown in A) and B), respectively, with different colors for different modes. Zero values of $\alpha$ are included as the limiting cases, overlaid on the logarithmic scale to show the trace modes SR and CR.


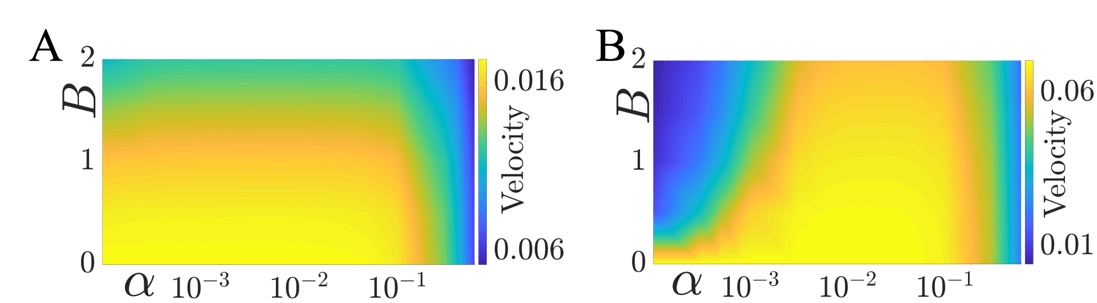


Figure S3.

Progressive velocity of the sperm head center trajectory in the lab frame, changing with waveform asymmetry (*B*) and rotation amplitude (
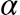
). A) and B) Results for the
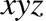
-model using
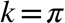
 and
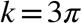
, respectively. The zero origins pertain to the vertical axis of *B*, and not the logarithmic horizontal axis representing
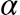
---zero is not displayed in horizontal axis. The linear speed is dimensionless and in units of flagellar arc length/ beat cycle.


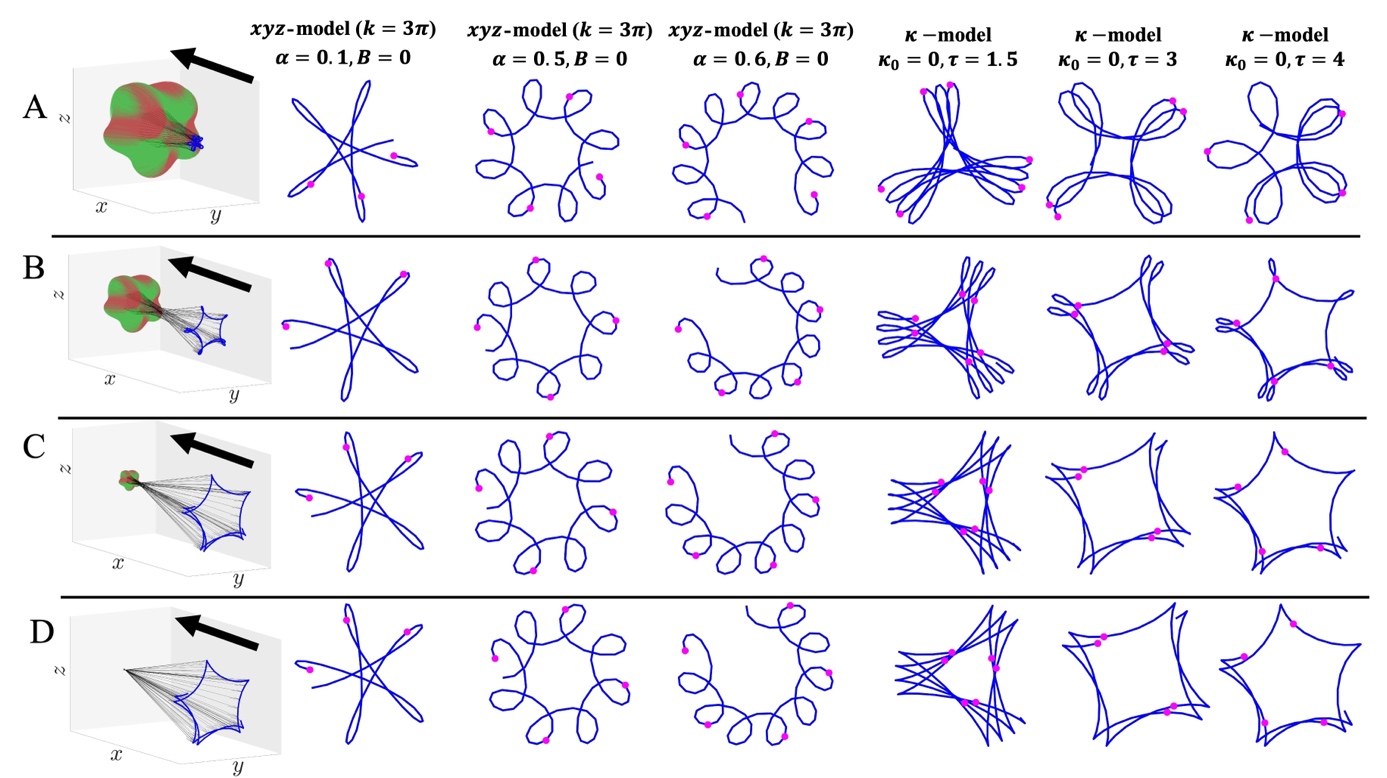


Figure S4.

Trajectories of sperm head longitudinal axis. A) The first figure on the left: schematic illustrating the trajectory along the negative side of the sperm head's long axis $\boldsymbol{\xi}_{3}$ (from the head center to the flagellum), with the sperm swimming direction indicated by a black arrow. The rest figures: the trajectories of the head's longitudinal axis, at a distance from the head center, for different virtual sperm models. The parameters of the virtual sperm models are listed at the top. Magenta points along the sperm trajectories are placed at intervals corresponding to the waveform beat cycle, indicating the synchrony between the flagellar beating and the helical path. B)-D) Same results but for different head axis lengths. From A) to D), the dimensionless axis lengths are 0.1, 0.3, 1 and 10, respectively. For the displayed
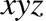
-model, the effects of axis length on the trace patterns are marginal, without altering the main trace feature of loops. However, for the displayed
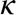
-model, the loop feature degenerates into cusps as the axis length increases.

**
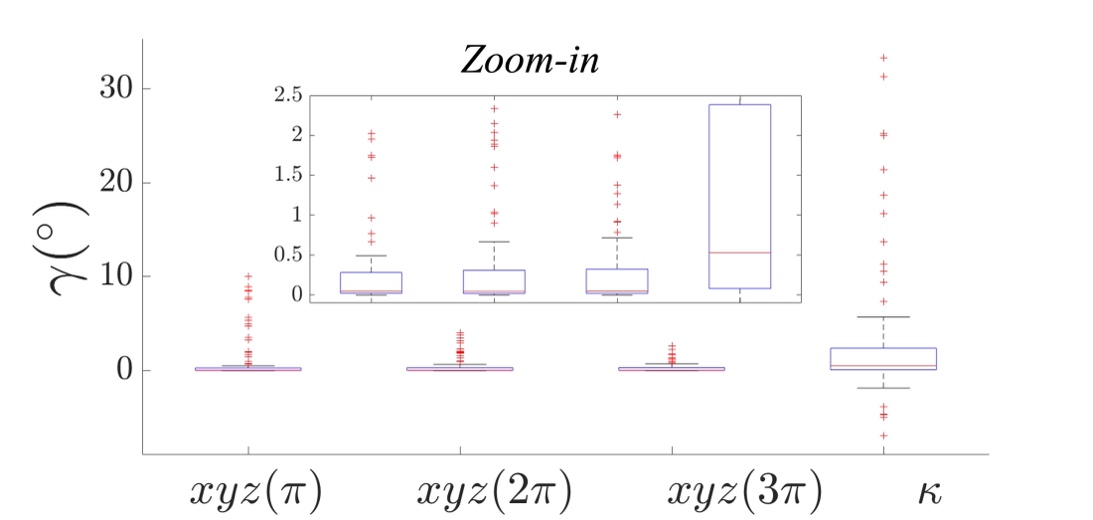
**

Figure S5.

Statistics of the angle
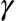
 between sperm head precession axis and the progressive axis of the corresponding swimming trajectory (see Figure 1 in the main text). All virtual sperm models are analyzed, including
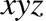
-models (
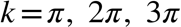
) and
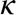
-model. Zoom-in inset enlarges the statistics detail to show that the medians and interquartile ranges of
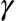
 are close to 0.


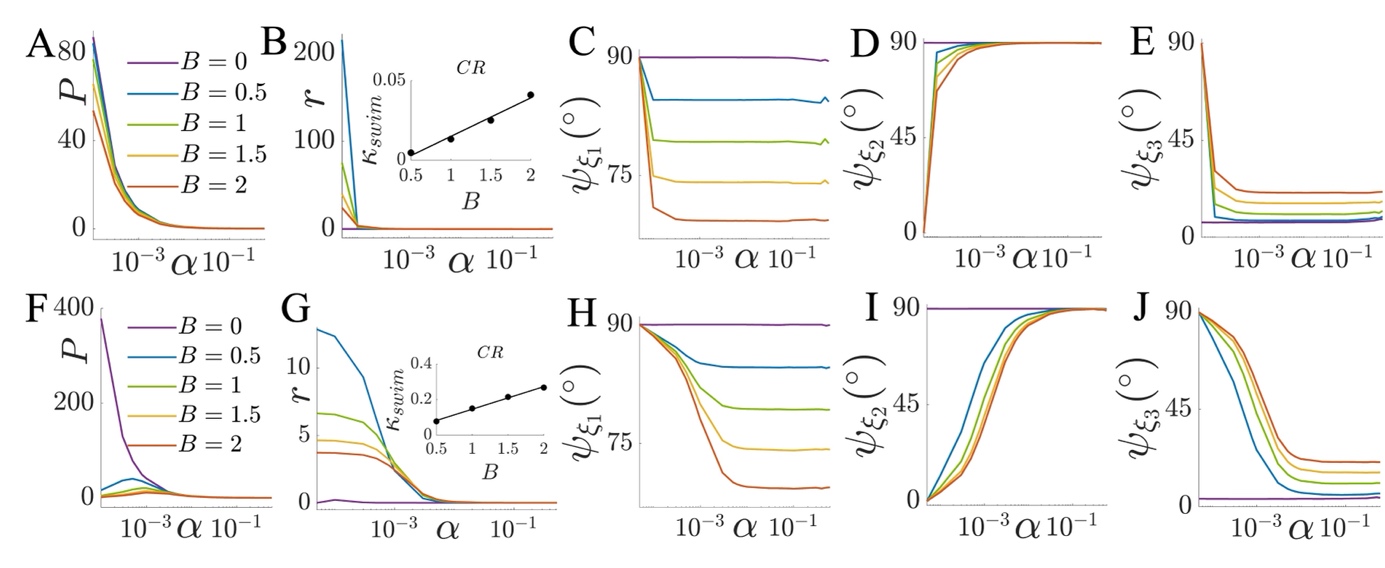
Figure S6.

Parameterization of sperm locomotion, changing with flagellar asymmetry and rotation amplitude. A) and B) Longitudinal (*P*) and transverse (*r*) envelopes of the aligned head trajectory subordinate to the
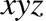
-model using
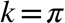
. The inset is for the CR mode, with $\alpha=0$, and shows a linear relationship between trajectory curvature
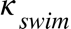
, inverse of the average path radius, and waveform asymmetry *B*. C)-E) Tilt angles of sperm head orientation orbits, subordinate to the
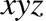
-model using
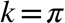
. From left to right are the average angles for the orthogonal vectors
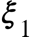
,
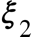
 and
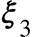
, in sequence. F)-J) Same results but for the
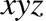
-model using
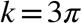
. Note that the zero origins pertain to the vertical axis, and not the logarithmic horizontal axis representing $\alpha$---zero is not displayed in horizontal axis.


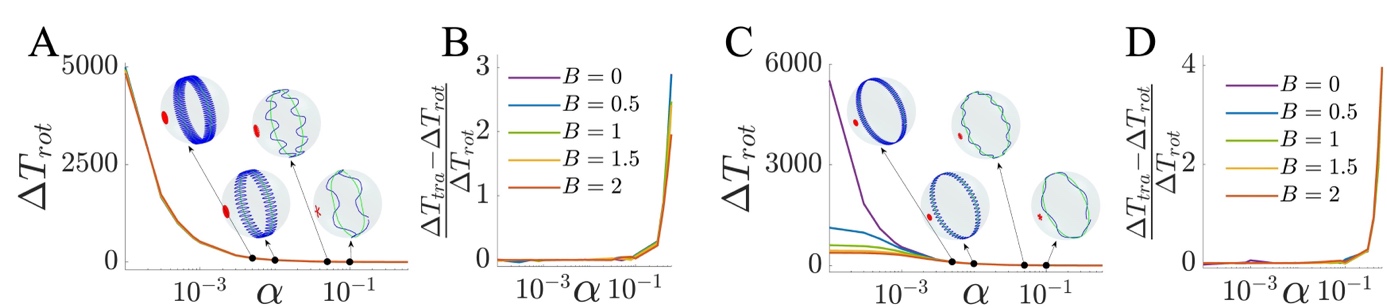


Figure S7.

Periods of sperm cyclic movements, rotation of head orientation orbits (
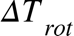
) and revolution of head center trajectory (
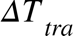
), in function of waveform asymmetry and out-of-plane component. A) Orientation orbit periods of the
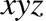
-model using
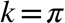
. Insets exemplify the orientation orbits within one
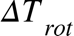
, with their spatial frequency decreasing when
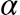
 increases. B) Relative deviation between the trajectory revolution cycle and the orientation rotation period, for the
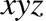
-model using
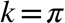
. C)-D) Same results but for the
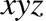
-model using
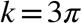
.


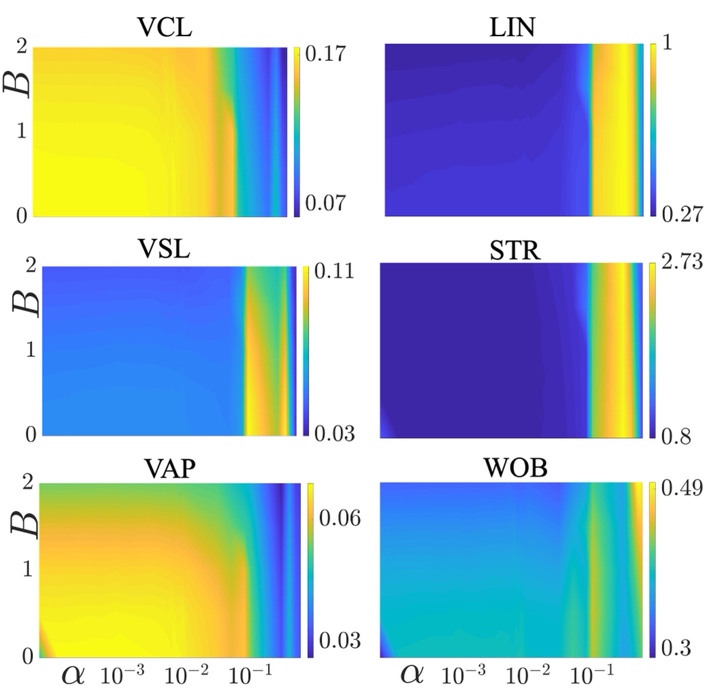


Figure S8.

Kymographs of 3D computer-assisted sperm analysis parameters, based on the
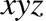
-model using
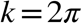
. A) Curvilinear velocity (VCL), B) straight-line velocity (VSL), C) average-path velocity (VAP), D) linearity (LIN), E) straightness (STR) and F) wobble (WOB), varying with
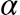
 and *B*. The linear speeds are dimensionless and calculated within
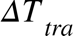
, in units of flagellar arc length/ beat cycle.

Movie S1.

Transient movements of sperm head center in the lab frame, for
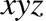
-model. Instantaneous swimming trajectories of the 6 trace modes, SR, CR, HR, TR, SS and HL, are shown in this video, and the characteristic of wiggling within one beat cycle is emphasized by red curves.

Movie S2.

Transient movements of sperm head center in the lab frame, for
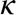
-model. Same as Supplementary Movie 1, except for
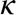
-model.

Movie S3.

The forward direction of free-swimming sperm is regulated by waveform rotation amplitude and asymmetry, based on the
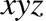
-model using
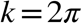
. The lab trajectories of sperm head center and corresponding forward directions, represented by arrows, are shown, with the waveform rotation amplitude **
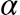
** changing from 0.0001 to 0.003 and the asymmetry factor *B* changing from 0 to 2.

Movie S4.

Sperm behaviors vary with waveform rotation amplitude, based on the
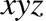
-model using
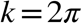
, *B*=2. Waveform pattern in the body frame, head center trajectory in the lab frame and the orientation orbits of head rotation are displayed together, with the combined results changing with different **
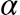
** values. The trajectory revolution characteristic is marked by a magenta curve connected at intervals of waveform beat cycle, and the forward swimming direction is indicated by an arrow, which changes synchronously with the head orientation on the sphere.

Movie S5.

Sperm behaviors vary with waveform rotation amplitude, based on the
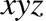
-model using
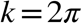
, *B*=0. Same as Supplementary Movie S4, except for *B*=0.

Movie S6.

Simultaneous sperm motions based on the
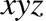
-model using
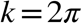
,
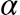
=0, *B*=2, including flagellar beating in the body frame, resultant movements of sperm swimming and head rotation in the lab frame. The cell trajectory in the lab frame induced by the planar waveform is curvilinear, and the trajectory revolution characteristic is marked with a magenta curve connected at intervals of waveform beat cycle. Head orientation during swimming is indicated by the basis vectors $\boldsymbol{\xi}_{1,2,3}$, and its rotational movement is condensed on a unit sphere.

Movie S7.

Simultaneous sperm motions based on the
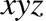
-model using
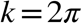
,
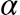
=0.05, *B*=2. Same as Supplementary Movie S6, except for **
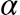
**=0.05. The trace of the mid-flagellar point in the body frame is quasi-planar, but the mid-flagellar point trajectory in the lab frame shows a more complex pattern, and the trajectory revolution characteristic is marked by a magenta curve connected at intervals of waveform beat cycle.

Movie S8.

Simultaneous sperm motions based on the
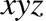
-model using
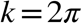
,
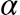
=0.5, *B*=2. Same as Supplementary Movie S6, except for
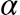
=0.5. According to the helicoid waveform, the trace of the mid-flagellar point in the body frame forms an ellipse-like shape, but the mid-flagellar point trajectory in the lab frame shows a more complex pattern, and the trajectory revolution characteristic is marked with a magenta curve connected at intervals of waveform beat cycle.

Movie S9.

Simultaneous sperm motions based on the
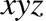
-model using
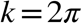
,
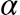
=-0.5, *B*=2. Same as Supplementary Movie S6, except for
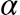
=-0.5. According to the helicoid waveform, the trace of the mid-flagellar point in the body frame forms an ellipse-like shape, but the mid-flagellar point trajectory in the lab frame shows a more complex pattern, and the trajectory revolution characteristic is marked with a magenta curve connected at intervals of waveform beat cycle.

References

1. Daloglu M U, Luo W, Shabbir F, et al. Label-free 3D computational imaging of spermatozoon locomotion, head spin and flagellum beating over a large volume[J]. Light: Science & Applications, 2018, 7(1): 17121-17121.
2. Pesch S, Bergmann M. Structure of mammalian spermatozoa in respect to viability, fertility and cryopreservation[J]. Micron, 2006, 37(7): 597-612.
3. Walker B J, Phuyal S, Ishimoto K, et al. Computer-assisted beat-pattern analysis and the flagellar waveforms of bovine spermatozoa[J]. Royal Society open science, 2020, 7(6): 200769.
4. Magdanz V, Vivaldi J, Mohanty S, et al. Impact of segmented magnetization on the flagellar propulsion of sperm‐templated microrobots[J]. Advanced science, 2021, 8(8): 2004037.
5. Magdanz V, Khalil I S M, Simmchen J, et al. IRONSperm: Sperm-templated soft magnetic microrobots[J]. Science advances, 2020, 6(28): eaba5855.
